# Economic policy uncertainty and the dynamics of healthcare expenditure in China

**DOI:** 10.3389/fpubh.2026.1788445

**Published:** 2026-04-21

**Authors:** Yuan Sui, Lantong Zhang, Shuyu Han, Qianrui Yun

**Affiliations:** 1 School of Taxation, Jilin University of Finance and Economics, Jilin, Changchun, China; 2 School of Economics, Jilin University, Jilin, Changchun, China; 3 Jilin Academy of Social Sciences, Changchun, Jilin, China

**Keywords:** dynamics, economic policy uncertainty, healthcare expenditure, regional heterogeneity, SV-TVP-FAVAR model

## Abstract

This study examines the dynamic impact of Economic Policy Uncertainty (EPU) on China’s healthcare fiscal expenditures, focusing on the rational allocation of public funds under macroeconomic fluctuations. The study utilize the SV-TVP-FAVAR model to analyze quarterly national data (2007–2025) and inter-provincial data (2009–2019), overcoming traditional static model limitations. EPU shocks exhibit significant time-variability: a negative “squeezing” effect during the 2008 financial crisis but a counter-cyclical expansionary effect during the 2020 COVID-19 pandemic. Regional fiscal strength moderates these impacts. Findings suggest that fiscally robust regions show resiliency through counter-cyclical spending, whereas fiscally fragile areas exhibit pro-cyclical tightening. Policy recommendations focus on long-term healthcare investment mechanisms and optimized transfer payments.

## 1 Introduction

In China’s constantly developing medical and health system, financial expenditure is a key tool to ensure public health and promote social equity, which has a profound impact on social welfare and stable economic development. At the same time, the global economic pattern is undergoing an adjustment. As the main driver of macroeconomic fluctuations, EPU has become the focus of macroeconomic research. Internal and external economic uncertainty shocks impact the effectiveness and adjustment trajectories of macroeconomic policy responses across the globe (1). The increase in the EPU level will inhibit the investment decision-making of enterprises through the “wait-and-see effect” mechanism, that is, enterprises will postpone capital expenditure to wait for clearer policy signals (2). The research by Bai et al. (3) expands this understanding in the spatial dimension. Through empirical analysis, they point out that economic policy uncertainty in China has a spatial spillover effect on local government healthcare expenditures. Subsequent scholars further confirmed that this uncertainty will also affect household consumption behavior through the adjustment of income expectations (4), and change the structure and scale of government expenditure through fiscal sustainability expectations (5, 6). The healthcare system in China has a broad integration of health care delivery systems within the broader fiscal system of China. The responsibility to provide public health services and medical resources rests primarily on local government authorities. However, there tends to be a structural mismatch between local governmental fiscal capacity and the rigid nature of the expenditure mandate. Therefore, the health care fiscal expenditure system performs a dual macro- and micro-control function, while at the same time serving the strategic goals of the country, such as “Healthy China 2030.” As result, the funding for the health care fiscal expenditure system is quite sensitive to the local fiscal health as well as external macroeconomic shocks. For example, fiscal stimulus after the 2008 global financial crisis focused on infrastructure construction, while it shifted to public health during the COVID-19 pandemic in 2020. Besides, recent literature in the post-pandemic era increasingly emphasizes the critical need for fiscal resilience in healthcare systems to withstand complex macro-shocks (7). De Lucchi (8) finds that the U. S. experienced a V-shaped recovery with robust broad money expansion during COVID-19, in stark contrast to the L-shaped post-GFC trajectory, highlighting how crisis-specific shocks shape distinct fiscal-monetary response dynamics. To capture these complex dynamics accurately, it is necessary to fouces on the improvement of the vital application of time-varying parameter approaches to evaluate the non-linear responses to uncertainty shocks (9).

At present, the global landscape is becoming increasingly complex, driving EPU to continuously elevated levels. Simultaneously, the world has recently experienced massive public health emergencies, which have inflicted unprecedented shocks on medical and healthcare systems globally. Against this turbulent backdrop, Chinese provincial administrations are also facing dramatic structural pressures resulting from diminished tax revenues and rigid social welfare spending demands. Therefore, there is a strong and urgent motivation to deeply investigate exactly how healthcare fiscal expenditure reacts when confronted with these severe EPU shocks. With all this in mind, research into the dynamic connection between EPU shocks and healthcare fiscal expenditure has transitioned from a theoretical exercise to an enormous practical need for building resilient public health systems and delivering equitable health services for all citizens.

The literature has explored the effect of EPU on the actions of macroeconomic entities; however, there are gaps in the literature analyzing the extent of the transmission to public health expenditure at a fiscal level systematically. (1) In researching the total scope, many studies examined only total fiscal expenditures or productive investments in infrastructure and education (10). The present literature typically combines public service with social security and avoids demonstrating the urgency of increased investment needed in healthcare during periods of systemic crisis (11). (2) The bulk of current literature employs highly static or fixed-parameter VAR modeling techniques. Little to no contemporary literature highlights the fact EPU shocks will have time-varying, nonlinear-transmitted characteristics across various economic cycles; hence the direction and magnitude of the fiscal responses to EPU shocks can dramatically alter due to the characteristics of the crisis involved (12). (3) In recent years, scholars have increasingly focused on how local fiscal constraints affect public spending, particularly highlighting expenditure preferences under the tax-sharing system (13). However, research remains limited on whether these constraints lead to pro-cyclical or counter-cyclical responses in specific regions when facing economic policy shocks.

The theoretical foundation of this study is systematically constructed upon the intersection of macroeconomic transmission theory, the theory of fiscal decentralization, and the hierarchical theory of local government expenditure. When uncertainty increases in the economy, local businesses will be less willing to invest in the future until they see how events unfold (2). As a consequence, local business investment and consumer spending decrease, thus trailing negatively on overall economic conditions and reducing local revenue from taxes. Due to the system of decentralized fiscal management in China, these local revenue shocks interact with vertical fiscal imbalances; therefore, local governments will have severe limitations on their budgets. According to the theory of hierarchical fiscal transfer expenditures, local governments facing a fiscal crisis behave very differently based on the type of fiscal shock that is occurring. During a broad fiscal crisis where general economic activity declines (i.e., the financial crisis of 2008), governments’ priority will be to invest in productive economic activity so they can recover economically and they will deem the cost of providing healthcare services to be, relative to the overall economic situation, a cost that can be compressed implicitly resulting in a pro-cyclical crowding out. On the other hand, when a systemic threat to public health exists (i.e., the COVID-19 pandemic of 2020), as a result of the nature of the crisis, healthcare will be viewed as a firm non-negotiable priority for state security requiring a counter-cyclical fiscal expansion to provide the necessary funding. In addition, the actual capacity to deal with these fiscal shocks will vary due to the particular region’s level of fiscal capacity at the time of incurring the shock, thus determining the degree to which a region absorbs or amplifies these uncertainty shocks. The comprehensive theoretical model will provide a framework for the subsequent analysis of empirical data.

By highlighting the gaps between previous studies and what has not yet been studied, the overall objective of this research is to systematically investigate the dynamic, time-varying effects of economic policy uncertainty on healthcare fiscal expenditures in China, as well as to identify the underlying transmission mechanisms of economic policy uncertainty through different types of crises and levels of regional fiscal strength. To achieve these objectives, we will use national quarterly data (2007–2025) and provincial (sub-national) level data (2009–2019) to build a SV-TVP-FAVAR Model. We will obtain common core factors from a large number (*n* = 98) of macroeconomic variables; In addition to comparing the fundamentally different impact trajectories of economic policy uncertainty during the 2008 Global Financial Crisis and the 2020 COVID-19 pandemic, we will also assess the heterogeneous responses of economic policy uncertainty on healthcare fiscal expenditures based on level of regional fiscal strength.

The original aspects and main contributions of this study to the existing literature are threefold: (1) The use of the SV-TVP-FAVAR methodology and three-dimensional impulse response functions allows this research to address the limitations of traditional static models by examining how the extent and duration of EPU shocks change dynamically throughout the different phases of the economic cycle rather than remaining constant. (2) The research finds that the impact of EPU on the economy differs based on the specific nature of the shock. For example, EPU was pro-cyclical in reducing economic activity during the 2008 Global Financial Crisis but the opposite (counter-cyclical) during the 2020 COVID-19 pandemic. (3) This research spatially refines current literature on the subject (3) by highlighting regional fiscal capacity as a significant moderating factor, thereby contributing to the literature on fiscal decentralization. The research highlights how regions that experience fiscally robust economic conditions experience counter-cyclical resiliency to EPU shocks; conversely, fiscally weak areas are pro-cyclical vulnerable to EPU shocks and demonstrate how uncertainty exacerbates regional public health disparities.

## 2 Method

### 2.1 Model specification

In order to systematically examine the dynamic and complex impact of EPU on China’s medical and health financial expenditure, this paper draws on the advanced methodology proposed by Jin and Zhang (14) to construct a random fluctuation time-varying parameter factor enhancement vector self-regression (SV-TVP-FAVAR) Model. The model combines factor augmentation, time-varying parameters, and stochastic volatility in one framework. There are three reasons this was the best model to implement when compared to the traditional VAR models: (1) The FAVAR mechanism identifies the key macroeconomic elements that exist across high dimensional data and provides a means to avoid dimensionality issues. (2) The nature of the model where the coefficients represent a time-varying parameter allows for the coefficients of the model to change over time. (3) The SV specifications of the model allow for models to be estimated accounting for the heteroskedasticity and clustering of shocks during times of crises so that we do not underestimate the effect of uncertainty during these turbulent periods in time (14, 15). The model integrates three key methods of factor enhancement, time-varying parameters and stochastic fluctuations, effectively overcoming the inherent limitations of the traditional econometric model, so as to more accurately capture the intricate nonlinear dynamic relationship between macroeconomic uncertainty and fiscal behavior.

First of all, the model introduces the factor enhancement mechanism, which provides an effective solution to solve the “dimensional defects” that puzzle the traditional vector autoregression (VAR) model. In order to solve this problem, the SV-TVP-FAVAR framework adopted in this paper extracts a small number of potential common factors from the high-dimensional data set containing 98 macroeconomic indicators. This setting retains the system information contained in the high-dimensional data set to ensure that the analysis reflects the broader macroeconomic environment, while maintaining the controllability of the number of variables, so as to ensure the estimation efficiency and stability of the model.

Secondly, the model adopts the time-varying parameter (TVP) setting, which is crucial to effectively capture the dynamic evolution of China’s economic structure and policy environment. It helps to identify structural fractures and transformations, and reflects changes in the intensity and even direction of the impact that may occur in different periods.

Finally, the model introduces the stochastic volatility (SV) setting, so that the framework can adapt to the heterovariance in the economic system. Macroeconomic time series data often show the phenomenon of volatility aggregation, that is, after the high volatility period is the high volatility period, and the calm period after the calm period. Models that assume that the variance is fixed tend to underestimate the level of uncertainty during extreme events. In this study, the covariance matrix of the disturbance term is set to change with time. This setting enables the model to better capture the change of impact intensity in special periods. By considering these volatile fluctuations, the SV-TVP-FAVAR model significantly improves the accuracy of statistical inference and ensures that the pulse response analysis remains robust even in the case of non-constant variance.

To examine the dynamic impact of EPU on China’s healthcare fiscal expenditures, we first establish a basic VAR model (Equation 1):


$$y_{t}=b_{1}y_{t-1}+b_{2}y_{t-2}+\cdots +b_{p}y_{t-p}+\nu_{t}$$ (1)

Where, $y_{t}$ denotes the (l+1)×1 vector of observed variables, comprising China’s medical and health fiscal expenditure, the EPU index, and other key macroeconomic variables; $b_{i}(i=1,2,\cdots ,p)$ represents the (l+1)×(l+1) fixed coefficient matrices; $\nu_{t}∼N(0,\Omega )$ is the (l+1)×1 disturbance vector; and Ω is the (l+1)×(l+1) fixed covariance matrix. However, the model has two main limitations. First, the variable dimension usually does not exceed 20, which is difficult to accommodate many macroeconomic factors affecting medical and health expenditure. Second, the fixed coefficient and the fixed covariance matrix cannot reflect the time variation of the shock effect, so it needs to be further expanded.

In order to solve the problem of incorporating high-dimensional variables, this paper introduces the concept of dynamic factor enhancement. The n -dimensional observable macroeconomic variables $x_{t}$ (this study selects 98 variables, covering GDP, industrial value-added, completed fixed asset investment, demand deposit interest rates, etc.) are reduced to k unobservable common factors $f_{t}(k<<n)$ . These common factors and the original observed variables $y_{t}$ jointly constitute a new variable system ${y_{t}}^{'}=[{f_{t}}^{'},{z_{t}}^{'},m_{t}]$ , where $z_{t}$ represents the core observed variables and $m_{t}$ is the EPU index, thereby forming the FAVAR model. The factor extraction is realized through the following factor (Equations 2 and 3):


$$x_{\mathrm{it}}=˜\lambda_{i}^{f}f_{t}+˜\lambda_{i}^{z}z_{t}+˜\lambda_{i}^{m}m_{t}+u_{\mathrm{it}}$$ (2)

$$\;u_{\mathrm{it}}=\rho_{i1}u_{\mathrm{it}-1}+\rho_{i2}u_{\mathrm{it}-2}+\cdots +\rho_{\mathrm{iq}}u_{\mathrm{it}-q}+\varepsilon_{\mathrm{it}}$$ (3)

Where $x_{\mathrm{it}}$ is the i -th original macroeconomic variable; ${\tilde{\lambda }}_{i}^{f}$ 、 ${\tilde{\lambda }}_{i}^{z}$ 、 ${\tilde{\lambda }}_{i}^{m}$ are the loading coefficients for the common factors, core observed variables, and the EPU index, respectively; $u_{\mathrm{it}}$ is the residual term of the factor equation, which follows a q -order autoregressive process, with q being the autoregressive lag order; $\rho_{\mathrm{ij}}$ is the coefficient of the j -th lag (j=1,2,⋯,q) of the residual autoregressive process; $\varepsilon_{\mathrm{it}}∼N(0,\exp(h_{\mathrm{it}}))$ is the structural residual term with stochastic volatility, where $h_{\mathrm{it}}$ is the volatility factor characterizing volatility heterogeneity. By relaxing the assumption of fixed coefficient matrices in the basic VAR model and allowing the coefficient matrices to vary over time to capture the time-varying characteristics of EPU shocks, the TVP-FAVAR model is obtained (Equation 4):


$$y_{t}=b_{1t}y_{t-1}+b_{2t}y_{t-2}+\cdots +b_{\mathrm{pt}}y_{t-p}+\nu_{t}$$ (4)

Where $b_{\mathrm{jt}}(j=1,2,\cdots ,p)$ are the (m×m) time-varying coefficient matrices (where m=k+l+1 , representing the total dimension of the expanded variables, k is the number of common factors, and l is the number of core observed variables), which change with time t ; and $\nu_{t}$ is the disturbance vector. Furthermore, the time-varying coefficient matrices are vectorized as $B_{t}={\left(\mathrm{vec}{\left(b_{1t}\right)}^{'},\mathrm{vec}{\left(b_{2t}\right)}^{'},\cdots ,\mathrm{vec}{\left(b_{\mathrm{pt}}\right)}^{'}\right)}^{'}$ (where $B_{t}$ is formed by stacking the vectorized $b_{\mathrm{jt}}$ , and vec is the matrix vectorization operator stacking the matrix by columns into a vector). It is assumed that the parameter matrix follows the innovative random walk proposed by and Koop and Korobilis (16) in Equation 5:


$$B_{t}=B_{t-1}+J_{t}^{B}\eta_{t}^{B}$$ (5)

Where $J_{t}^{B}$ is an indicator variable ( $J_{t}^{B}=1$ indicates time-varying coefficients, $J_{t}^{B}=0$ indicates fixed coefficients); and $\eta_{t}^{B}∼N(0,Q_{B})$ is the parameter shock term, with $Q_{B}$ being the shock covariance matrix.

To further capture the volatility heterogeneity caused by sudden shocks in the macroeconomic system, the covariance matrix of the disturbance term in the TVP-FAVAR model is allowed to vary over time, i.e., $\nu_{t}∼N(0,\Omega_{t})$ . The time-varying nature of the covariance matrix is characterized through triangular matrix decomposition and stochastic volatility settings (Equations 6 and 7):


$$A_{t}\Omega_{t}{A_{t}}^{'}=\Sigma_{t}{\Sigma_{t}}^{'}$$ (6)

$$\Omega_{t}=A_{t}^{-1}\Sigma_{t}{\Sigma_{t}}^{'}A_{t}^{-1}$$ (7)

Where $A_{t}$ is an (m×m) lower triangular matrix with main diagonal elements equal to 1 (used for identifying structural shocks), and its non-main diagonal elements $\alpha_{t}={\left({\alpha_{j1,t}}^{'},\cdots ,{\alpha_{j(j-1),t}}^{'}\right)}^{'}$ follow a random walk process $\alpha_{t}=\alpha_{t-1}+J_{t}^{\alpha }\eta_{t}^{\alpha }$ (where $\eta_{t}^{\alpha }∼N(0,Q_{\alpha })$ ); $\Sigma_{t}=\mathrm{diag}(\sigma_{1t},\sigma_{2t},\cdots ,\sigma_{\mathrm{mt}})$ is a diagonal matrix containing the j -th diagonal element of $\Sigma_{t}$ , specifically the time-varying standard deviation $\sigma_{\mathrm{jt}}(j=1,2,\cdots ,m)$ , and $\log\sigma_{t}=\log\sigma_{t-1}+J_{t}^{\sigma }\eta_{t}^{\sigma }$ (where $\eta_{t}^{\sigma }∼N(0,Q_{\sigma })$ ).

Thus, the SV-TVP-FAVAR model is formally established, synthesizing factor augmentation, time-varying parameters, and stochastic volatility into a unified system. Constructed upon a dual foundation of factor equations and regression equations, this framework is capable of comprehensively characterizing the dynamic shocks of Economic Policy Uncertainty (EPU) on medical and health fiscal expenditure within a complex, high-dimensional macroeconomic variable system.

The comprehensive advantage of the SV-TVP-FAVAR model lies in its multi-functional functions: it not only maximizes the use of high-dimensional macroeconomic information and effectively avoids the omission of variable deviation, but also accurately captures the time-varying characteristics of variable relationships. By clearly considering the heterovariance of economic shocks, the model provides an ideal empirical framework for studying the dynamic impact of EPU on health expenditure. This specific model configuration is especially suitable for analyzing the complexity of financial expenditure behavior in China’s economic transformation and increasingly turbulent external environment. Therefore, it can provide policymakers with richer and more detailed spatial and temporal dimensional evidence, thus supporting more rigorous and effective policy evaluation.

### 2.2 Parameter estimation

In terms of estimation method, this paper uses the Markov chain Monte Carlo (MCMC) method under the Bayesian framework to sample the posterior distribution. Drawing on the pioneering research of Primiceri (17) and Koop and Korobilis (16), this study sets a random wandering process for time-varying parameters and specifies an appropriate *a priori* distribution for each parameter. The extracted common factors are regarded as unobservable potential variables and are included in the Bayesian estimation framework together with the time-varying coefficient ( $B_{t}$ ) and the time-varying covariance matrix parameters ( $\alpha_{t}$ , $\sigma_{t}$ ) of the model. This paper adopts the Gibbs sampling method for iterative estimation.

Specifically, the initial state of the time-varying coefficients is set as $B_{0}∼N(\hat{B},\hat{V})$ , where $\hat{B}$ represents the matrix of prior means (with the coefficient for the first lag set to 0.9 and the coefficients for the remaining lag terms set to 0), and $\hat{V}$ denotes the prior covariance matrix in the Minnesota form. The elements of the lower triangular matrix are specified as $\alpha_{0}∼N(0,4I)$ , and the volatility factors are specified as $\log\sigma_{0}∼N(0,4I)$ . For the shock terms of the covariance matrix, Inverse Wishart prior distributions are established as follows in Equation 8:


$$\left\{ \begin{array}{c} Q_{B}^{-1}∼W(0.005\times (\dim(B)+1)\times \hat{V},\dim(B)+1)\\ Q_{\alpha }^{-1}∼W(0.01\times (\dim(\alpha )+1)\times I,\dim(\alpha )+1)\\ Q_{\sigma }^{-1}∼W(0.0001\times (\dim(\sigma )+1)\times I,\dim(\sigma )+1) \end{array}\right.$$ (8)

Where W denotes the Wishart distribution (with the first parameter representing the scale matrix and the second parameter representing the degrees of freedom); and dim serves as the variable dimension operator, returning the length of the vector or the dimension of the matrix.

## 3 Empirical analysis

### 3.1 Variable selection and common factor extraction

The selection of the 2007–2025 period for the national-level analysis is primarily determined by data availability, as 2007 represents the earliest year for which consistent and high-quality quarterly records can be obtained. Extending the sample to 2025 ensures a sufficiently long time series that captures both the 2008 Global Financial Crisis and the COVID-19 pandemic, thereby enabling the SV-TVP-FAVAR model to effectively capture time-varying impulse responses under distinct crisis regimes. For the provincial-level heterogeneity analysis, the sample is restricted to the 2009–2019 period. This truncation is necessary for two main reasons. First, starting in 2020, several provinces discontinued the publication of key fiscal and economic indicators, leading to significant data gaps and inconsistencies. Second, the outbreak of the COVID-19 pandemic introduced extreme values and structural breaks in the data, which could distort the estimation of normal cyclical relationships and mask the underlying inter-provincial differences in fiscal behavior. Therefore, to ensure data consistency, reliability, and the validity of cross-provincial comparisons, we exclude post-2019 observations from the heterogeneity analysis.

All data used in this study are systematically extracted and compiled from the Wind Economic Database, which comprehensively aggregates official statistics originally published by the National Bureau of Statistics (NBS), the Finance Yearbook of China, public data releases from the Ministry of Finance (MOF), and local fiscal statistical materials.

In order to establish the overall credibility and validity of the empirical model, an extensive data pre-processing strategy was followed for the raw time series data. The first step consisted of converting the frequency of the raw Wind data into a uniform quarterly frequency using aggregation and alignment methods. The next step involved conducting seasonal adjustments on the macroeconomic variables that had significant seasonal patterns, in order to remove calendar effects and seasonal distortions. The last pre-processing step involved using the Augmented Dickey-Fuller (ADF) unit root test on the three different quarterly data sequences to ensure that they all met the basic statistical assumptions for carrying out time series analysis. Non-stationary variables found through this process underwent multiple differencing transformations until strict stationarity was attained, and only fully stationary time series data were eventually incorporated into the SV-TVP-FAVAR model. The descriptive statistical analysis for all variables used in this study is provided in Appendix Table 1.

#### 3.1.1 Explanatory variables

This study selects the Economic Policy Uncertainty Index as the main external shock variable. Early literature often used a single policy variable or an index based on the text of the news report (18), but its data source was single and not representative enough. It only relied on Hong Kong’s South China Morning Post (SCMP), an overseas English newspaper, which focused on issues related to Hong Kong and the mainland, the sensitivity to the internal policies of mainland China is low, and the translation and dissemination of policy terms in the English context are prone to information distortion. Therefore, this article draws on Huang and Luk (19), consisting of 74 quarterly observations matching the national sample period. Measured and constructed China’s EPU Index, which is optimized for China’s national conditions on the Baker framework, covering the data source to 10 mainland core Chinese newspapers to ensure geographical and policy representativeness, which is more in line with the characteristics of China’s policy environment. Compared with other methods, the EPU quarterly index measured by this method has stronger robustness and more reasonable fluctuation characteristics.

#### 3.1.2 Explained variable

The core dependent variable of this study is the financial expenditure on medical and health care. Specifically, we choose “health and family planning expenditure” (later renamed “health and family planning expenditure”) in the general public budget expenditure as the dependent variable (20). The data of this indicator comes from the China Fiscal Yearbook and the financial accounts of each province. This indicator directly reflects the scale of financial investment of local governments in the field of medical and health care, with the characteristics of authority, continuity and cross-regional comparability, and is an authoritative indicator for measuring the behavior of government health expenditure. Therefore, it provides an accurate quantitative tool for analyzing the dynamic impact of economic policy uncertainty on health expenditures. The national-level data volume consists of 74 quarterly observations (2007Q1–2025Q2).

#### 3.1.3 Common factor extraction

**Figure 1:**
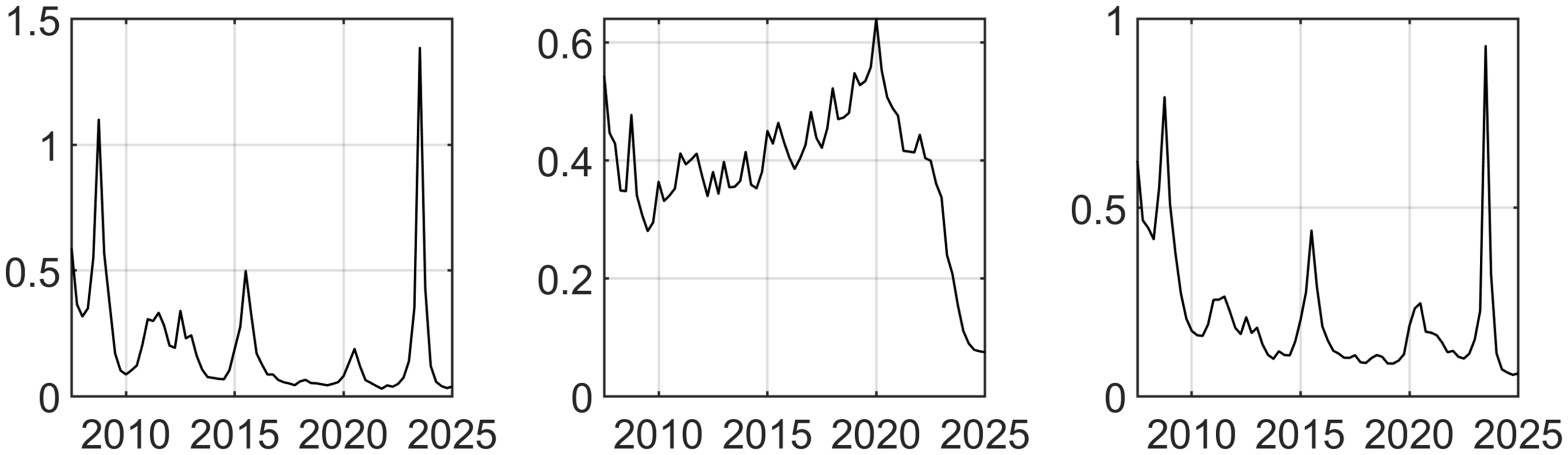
Time series of three common factors.

Macroeconomic system variables are sourced from the Wind Database, comprising 98 distinct macroeconomic indicators, extracting 3 common factors. In this study, three distinct common factors were extracted to analyze macroeconomic trends. By examining the trajectory of the posterior mean standard deviations of these factors, a clear cyclical pattern emerges. Most notably, the data shows that in 2008, 2015 and 2020, these factors showed violent fluctuations, with obvious fluctuation peaks. These fluctuation peaks correspond to major structural shocks and the period of economic transformation (Figure 1). 2008 was the first major fluctuation affected by the Global Financial Crisis; the external export demand looked at has declined drastically and domestic investor’s confidence has been negatively affected as well. The economy’s attempts of adapting to losing of Global-demand have caused the economic slowdown, this has had a negative impact on several of the economy’s main macroeconomic variables like the Industrial Added Value and the fiscal revenue that have been experiencing major Westernized Fluctuations. Around 2015 there was a second major peak in fluctuations, meaning the traditional growth engines are becoming weaker and deep structural changes have produced necessary but painful Short-Term shocks to the Economic System; there are also several financial instabilities occurring at the same time due to major turnaround in capital flows and also the changes to the Stock-Market and Reform of the Exchange Rate-Stage; overall all of these financial instabilities have caused increased uncertainty in Macro Economic indicators. Lastly, the last and in fact the most significant fluctuations were related to the Global COVID-19 pandemic. As an unprecedented public health emergency, the epidemic has had a comprehensive impact on China’s economic and social system. In terms of supply, production activities have stagnated due to extensive lockdown measures; in terms of demand, consumption has basically frozen. At the same time, the sudden surge in demand for medical resources forced the government to urgently increase financial expenditure on public health. In such an extreme situation, the complex connection between these variables leads to significant fluctuations in common factors.

In conclusion, these three common factors have successfully captured and explained the vast majority of the information contained in the 98 macroeconomic variables analyzed. Therefore, they can act as highly reliable carriers of macroeconomic information, laying a solid foundation for the subsequent model to accurately capture and analyze the dynamic impact of EPU.

### 3.2 Three-dimensional impulse response results

To deeply capture and analyze the dynamic evolutionary characteristics of the shocks generated by EPU on public health fiscal expenditure, this study employs a Three-Dimensional Impulse Response Function analysis. This advanced methodological approach is distinct in its ability to simultaneously visualize the complex interrelationships among three critical dimensions: the specific timing of the shock occurrence, the intensity of the response, and the duration of the lag periods. By integrating these dimensions into a single framework, the method systematically reveals the full landscape of the time-varying relationships between variables, offering a more granular view than traditional static models can provide.

**Figure 2:**
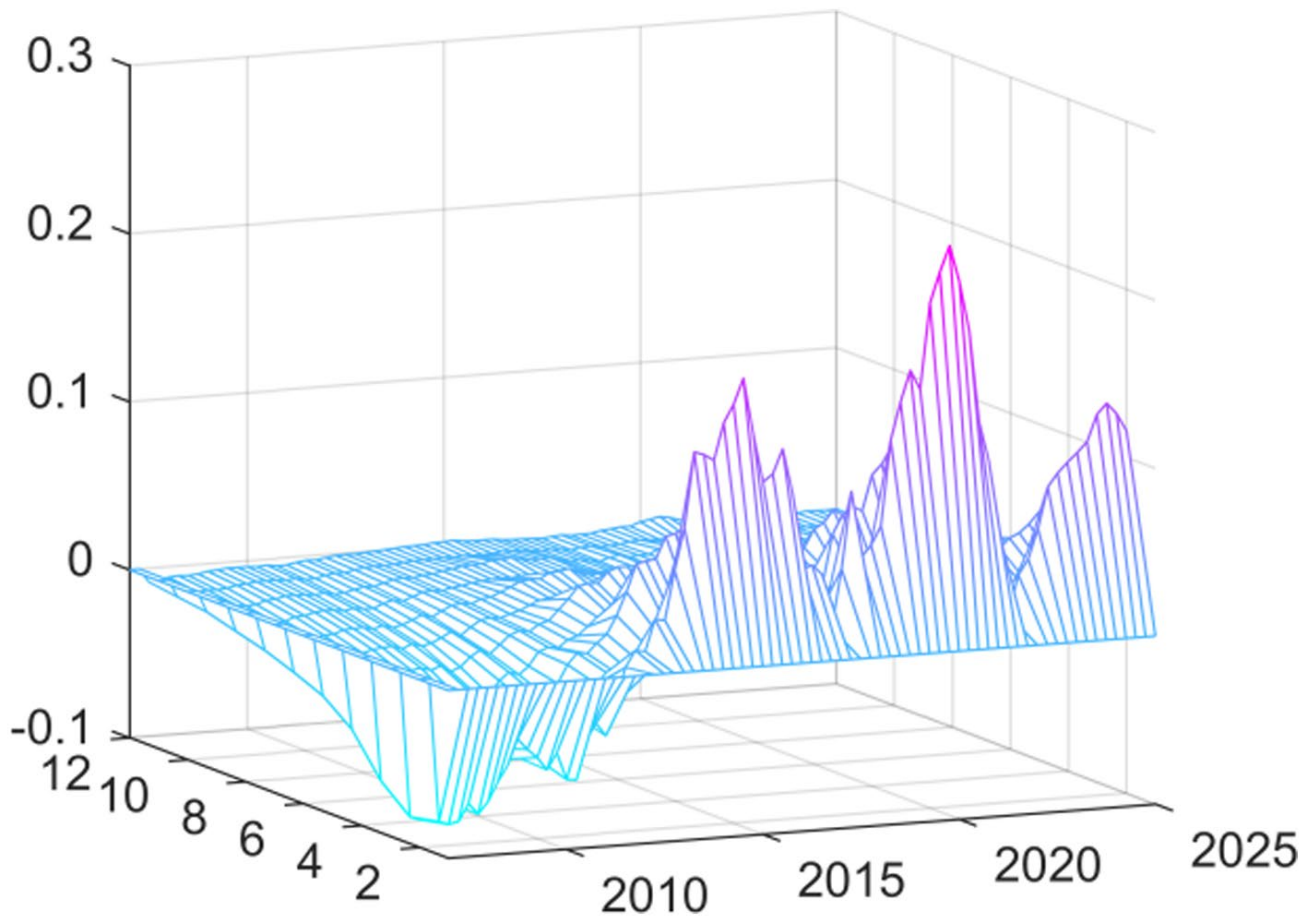
Three-dimensional time series surface plot of main regression.

Observing the three-dimensional impulse response plots, it becomes evident that the impact of EPU on medical and health fiscal expenditure exhibits pronounced time-varying characteristics and structural transformations. Overall, the shock effects demonstrate significant heterogeneity in terms of both intensity and direction across different historical periods. This variation underscores the systemic influence that shifting external environments and evolving policy objectives have on government fiscal expenditure behaviors. The response varies based on current macroeconomic conditions rather than being consistent. The findings reveal that there are two periods of exceptional volatility. The Global Financial Crisis generated the worst shock effect on the economy when EPU rose in relation to the economy. This occurred via different transmission channels: it decreased the real economy and reduced the government’s available financial resources. The downward pressure on these two channels correlated to a distinct “crowding out” or squeezing effect on public health expenditures. With increasing uncertainty came increasing fiscal constraints and the resulting reduction in funding for healthcare. The other period of high volatility and extreme fiscal uncertainty came at the start of the COVID-19 pandemic. Although the shock of the pandemic started with an extreme peak (Figure 2), it changed the relationship between policy uncertainty and spending during a public health crisis. During this time, uncertainty positively correlated with emergency fiscal input increases. The data show that while the government increased public spending on medical and health services due to COVID-19, increasing fiscal uncertainty was associated with a proactive increase in fiscal support for health services rather than decreasing it. Therefore, it appears that the adaptive nature of fiscal policy differs according to the type of crisis.

**Figure 3:**
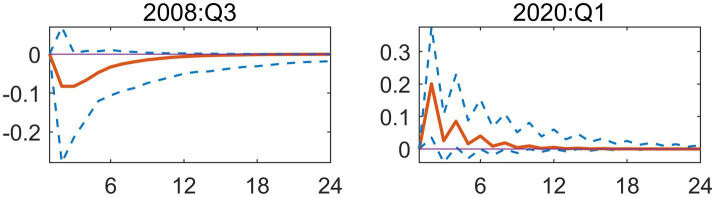
Impulse response function plot of main regression.

To further clarify transmission differences across scenarios, this paper analyzes two representative periods separately: Q3 2008 and Q1 2020. Q3 2008 marked the deepening phase of the global financial crisis, where a positive EPU shock triggered a significant negative short-term response in healthcare fiscal expenditures, reflecting procyclical tightening. The response curve reached its minimum value (approximately −0.1) in the second period, with a narrow 90% confidence interval (Figure 3). This indicates low estimation uncertainty for the shock effect during this period and the statistical significance of the negative impact. This closely aligns with the financial crisis period’s policy shift toward growth preservation, where healthcare spending faced suppression due to policy uncertainty. It reflects the traditional crisis response pattern where fiscal authorities treat healthcare expenditures as a cutback item to address macro-fiscal pressures. In Q1 2020, the outbreak of the COVID-19 pandemic triggered a positive EPU shock, resulting in a significant positive response in healthcare fiscal spending, demonstrating countercyclical expansion. The response curve peaked in Phase 2 (approximately 0.2) and gradually narrowed after short-term expansion, reflecting high uncertainty during the initial pandemic phase that stabilized as policies and containment mechanisms clarified (Figure 3). This indicates that amid major public health crises, increased EPU actually prompts governments to rapidly allocate resources to healthcare, implementing proactive expansionary fiscal policies to safeguard public health and emergency response capabilities.

To better understand differences in the response to EPU across different scenarios, this study will analyze two periods; Q3 2008 and Q1 2020, which represent different responses to and sources of inflation. The July–September months of 2008 were a critical moment, as this was when the global economic collapse began to spiral into a world-wide crisis. The effects of this crisis on healthcare expenditures can be seen, as the EPU increased significantly in this period, causing considerable decreases in short-term healthcare expenditures due to the negative effects of inflation on the economy. The overall response to EPU uncertainty in this period was near its lowest point, with a response curve of about −0.1 and a 90% confidence interval that was very tight, showing that the uncertainty around the effect of the shock on healthcare expenditures had been greatly reduced when the response curve reached this level. This is consistent with the economic uncertainty of the time, in that there were very few new investments or hiring of employees to address these problems, while at the same time there were large increases in unemployment, leading to cuts to healthcare expenses in response to the significant macro-fiscal pressures being placed on fiscal authorities in the US at that time. In the second period analyzed, Q1 2020, the COVID-19 pandemic created another major source of causes for increasing economic uncertainty, and therefore caused another large, positive increase in EPU, which resulted in a large increase in fiscal expenditures for healthcare in a counter-cyclical manner. The peak in fiscal spending due to this increase in EPU was approximately 0.2 and began to decline rapidly by the end of March 2020, as policies, containment mechanisms, and other factors helped stabilize the influence of COVID-19 on fiscal policies (Figure 3). This suggests that the increased uncertainty of economic policies during major public health crises has actually prompted the Government to rapidly allocate resources to the health-care sector and implement active expansionary fiscal policies to guarantee public health and emergency response capabilities.

At the same time, we are going to explain the different transmission mechanisms we have in various macroeconomic contexts and for this we have to focus on very few instances in time, in this case we will choose two: the third quarter of 2008 and the first quarter of 2020. These two instances are paradigmatic for explaining how the relationship between uncertainty and fiscal actions evolves, depending on the type of crisis. The first of them, Q3 of 2008, relates to the intensification of the Global Financial Crisis. A Positive shock in EPU during this window led to a very short-term negative response of medical and health fiscal expenditures, which is a prototypical contraction, pro-cyclically. The response curve of this contraction declines and reaches its floor during the second window of time, to a value of, say, approx. –0.1. The relatively narrow 90% confidence interval for this window of time is a very important high-order statistics observation-this means that, regarding the shock effect, the uncertainty in the estimation was very low, and the negative shock was significant (Figure 3).

From an economic perspective, this importance has its roots in the way the Government altered their strategy during the financial crisis. The Government began placing more emphasis on growth stabilization and economic rescue packages, while at the same time focusing their attention on fiscal resources. As a result, Economic Policy Uncertainty resulted in an adverse effect on Medical/Health Expenditures due to uncertainty in policies affecting economic growth and recovery. Therefore, during periods of economic crisis, Local Fiscal Authorities perceived that Medical/Health Expenditure could be viewed as discretionary or “cuttable” items, which local authorities could cut back on in order to relieve some of the macroeconomic fiscal stress. The second node, the first quarter of 2020, represents a contrast to the previous node in that the COVID-19 pandemic created a positive shock to Economic Policy Uncertainty, which in turn resulted in increasing Medical/Health Expenditures in an expansive counter-cyclical pattern. The initial response from Medical/Health Expenditure produced a peak in the response curve of approximately 0.2 in period two, followed by a tapering of the initial surge to a peak due to the accumulation of Economic Policy Uncertainty during the initial phase of the COVID-19 Pandemic, and subsequently stabilizing as policy initiatives and methods for preventing the onset of the virus became more clear and effective (Figure 3).

This change in course shows how the nature of the crisis significantly modifies the policy response. An increase in EPU did not result in austerity in the particular setting of a significant public health emergency. Rather, it served as a driving force behind the government’s quick allocation of funds to the health and medical fields. The government sought to ensure public health security and preserve strong emergency response capabilities by enacting active, expansionary fiscal policies. Therefore, in the 2020 scenario, uncertainty prompted immediate fiscal mobilization to safeguard the economy’s foundation, in contrast to the 2008 scenario where uncertainty led to fiscal retrenchment in social sectors.

Synthesizing the empirical evidence from both the three-dimensional impulse response functions and the analysis of typical historical periods yields a critical conclusion regarding the relationship between EPU and public health fiscal expenditure. The analysis demonstrates that the impact of EPU is not uniform; rather, both the directionality and the magnitude of its influence are fundamentally contingent upon the specific nature of the dominant crisis prevailing at the time.

There is a distinct heterogeneity in the government’s fiscal response mechanisms across different categories of extreme events. In the context of a financial crisis, such as the 2008 recession, the reaction of fiscal expenditure tends toward contraction. This suggests a scenario where economic constraints and declining revenues compel a squeezing effect on health spending. Conversely, during a public health crisis like the COVID-19 pandemic, the response mechanism shifts dramatically toward expansion. In these instances, high uncertainty acts as a catalyst for increased government intervention. This divergence highlights that fiscal policymakers adopt adaptive strategies, prioritizing fiscal consolidation during general economic downturns while pivoting to aggressive resource allocation and emergency funding during existential public health threats.

### 3.3 Robustness test

In order to strictly ensure the reliability of the conclusions obtained from the reference regression, this study conducted a series of robustness tests. The empirical results consistently show that even if alternative measurement methods are used for key variables, the impact pattern of EPU on the investment in the health sector over time is still highly consistent with the main regression results. This consistency strongly confirms the robustness and validity of the core findings proposed in this study.

#### 3.3.1 Replacing the explained variable

In our initial analysis, we looked at “medical and health government spending” as the independent variable. The core difference between the two lies in their economic definitions and statistical scopes: while the original ‘fiscal expenditure’ strictly represents the government’s direct supply-side budgetary input, the replacement ‘total medical expenses’ indicator measures broader health investment from the perspective of the entire society’s consumption side—reflecting the actual realization of medical services including government, corporate, and individual expenditures.

**Figure 4:**
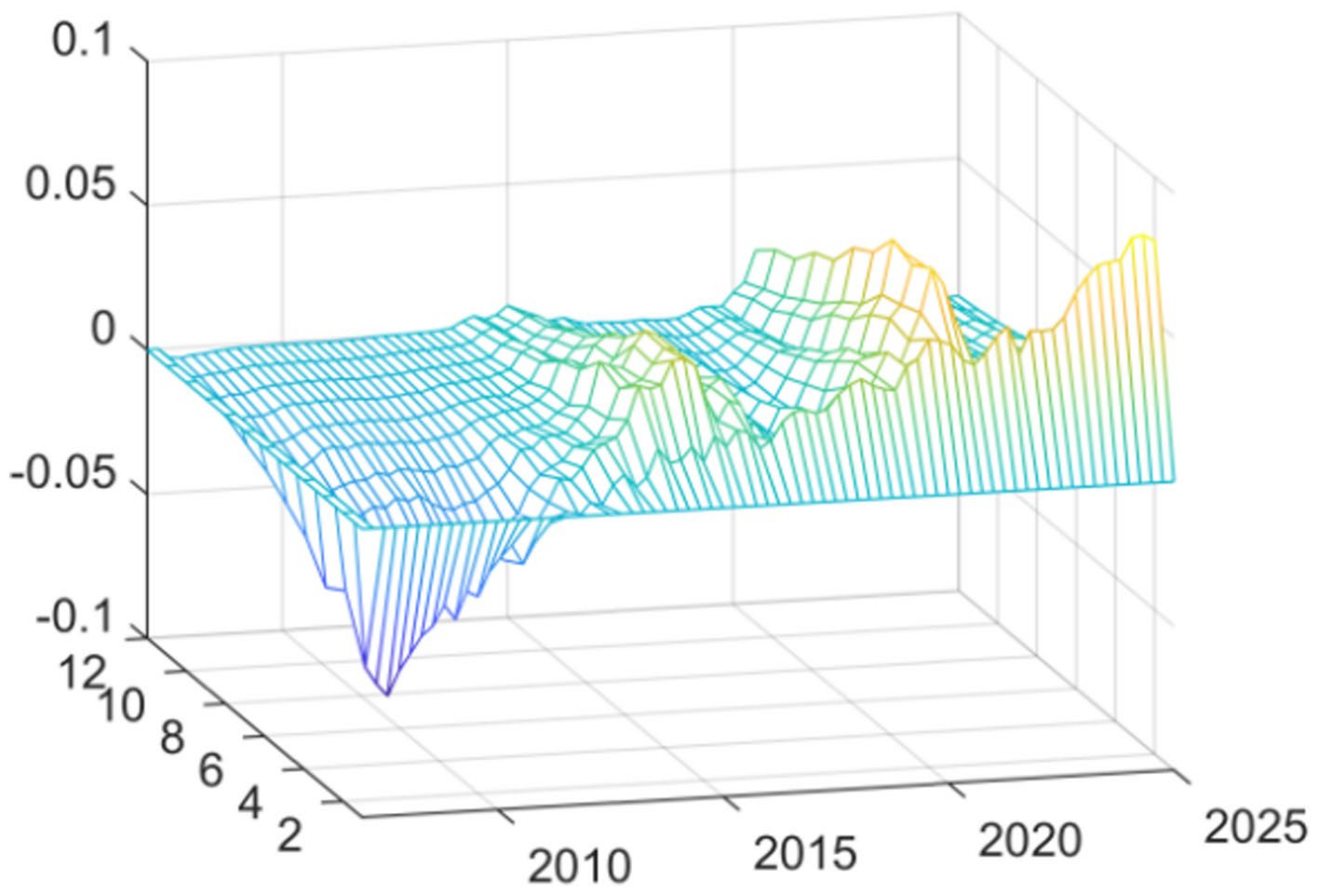
Replacing the explained variable: three-dimensional time series surface plot.

In this second set of results, we replaced this independent variable with “total medical costs” (available from the National Health Commission). The fiscal expenditure represents the input to the system from the government, while the total medical costs indicator represents the measurement of how much health services cost to use—indicating how much money was actually spent on getting medical services. As can be seen from Figure 4 the shapes of the curves resulting from the new independent variable are nearly identical to the results shown in Figure 2. The shapes and most features of the two curves are extremely similar. There was still a substantial inhibiting effect up until the time of the Global Financial Crisis in 2008. In contrast, at the onset of the COVID-19 pandemic in 2020, we see strong promoting effects.

**Figure 5:**
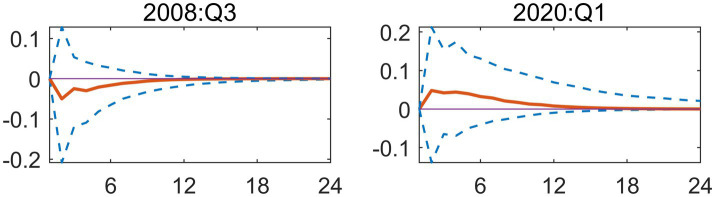
Replacing the explained variable: impulse response function plot.

The impulse response analyses shown in Figure 5 confirm this as well. The dynamic impacts of EPU shocks demonstrate similar directions, strengths and dynamics for both the financial crisis and the public health crisis as seen in the baseline model, suggesting that the nature of these effects does not depend on how the fiscal accounts are classified. Instead, the structural, cyclical, and change-driven impacts of EPU are experienced across the medical and health-related sectors on an equal basis. It is clear that the cumulative differences in EPU’s effects between the two different crises are not determined by how either the government’s fiscal inputs or society’s consumption, thus supporting the overall validity of the baseline results.

#### 3.3.2 Replacing the explanatory variable

To further verify the sensitivity of the study’s conclusions to the specific methodology used for measuring EPU, this paper conducts a second critical robustness check. In this analysis, the core explanatory variable—originally based on the Huang and Luk (19) index—is substituted with the internationally recognized and widely utilized EPU index constructed by Baker et al. (18).

The primary difference between these two indices lies in their underlying data sources and construction methodologies. While the original Huang and Luk index is specifically tailored to China using 10 mainland Chinese newspapers for enhanced domestic representativeness, the replacement Baker et al. index relies on a major overseas English newspaper (the South China Morning Post) and utilizes a globally standardized measurement framework.

**Figure 6:**
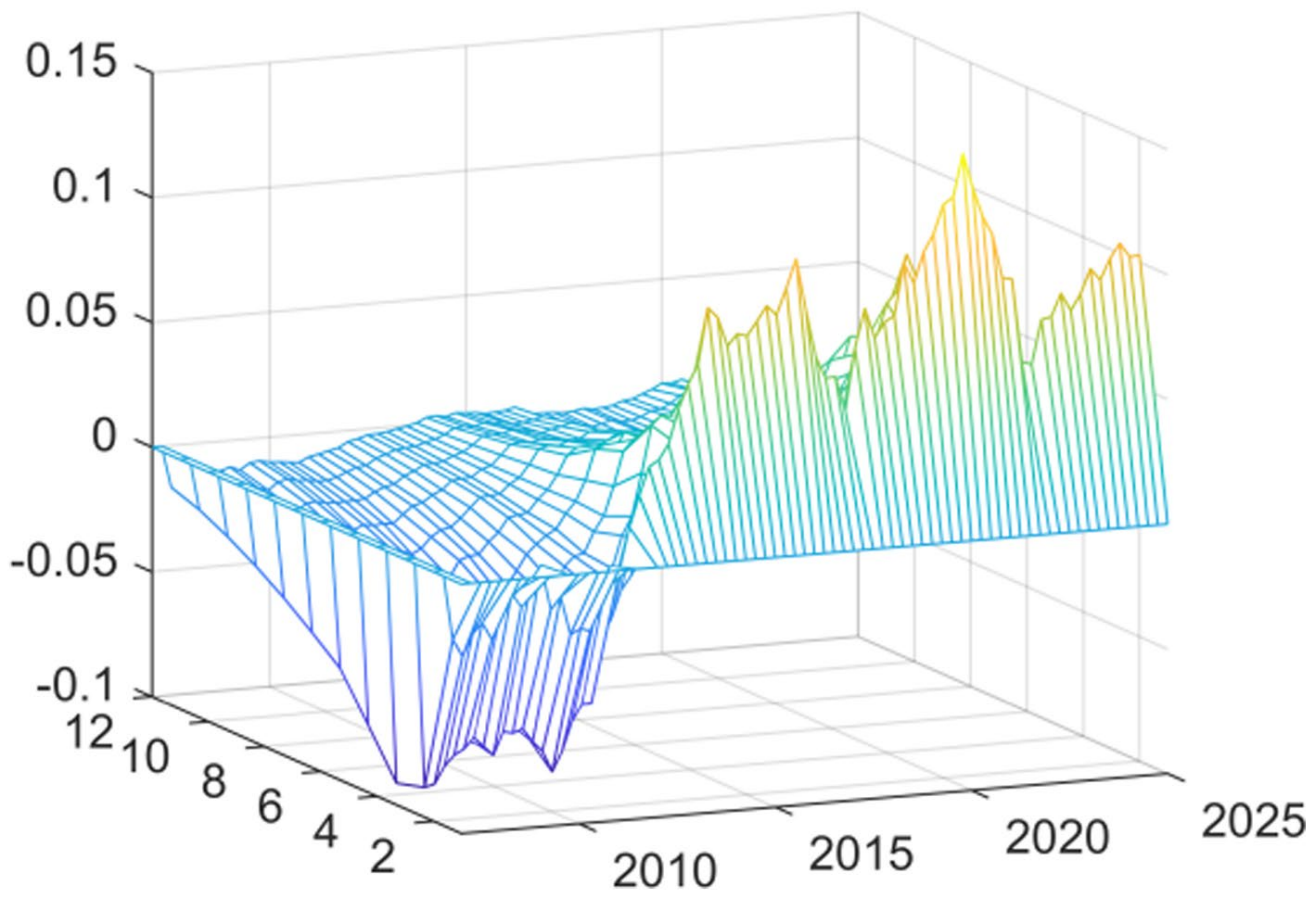
Replacing the explanatory variable: three-dimensional time series surface plot.

**Figure 7:**
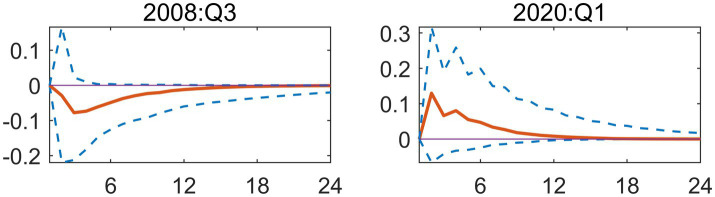
Replacing the explanatory variable: Impulse response function plot.

This second iteration of confirming the sensitivity of the conclusions to the measurement methodology of EPU supports the major finding of this paper, as it replaces the measuring instrument (based on (19)) for the independent variable (core explanatory variable) with a measuring instrument that has gained global acceptance and use (18). Figure 6 provides strong evidence to support the information presented above. The Baker et al. EPU index, while created using a different construction methodology than the baseline index, and based on very different underlying data sources, shows a similar change in how China’s overall health expenditures changed over time. Specifically, the basic shape of the curve showing the change in expenditure is the same; both the Baker et al. index and baseline index (shown in the impulse response graph; Figure 7) show a very large negative shock during the Global Financial Crisis (GFC) and a very large positive shock following the start of the COVID-19 pandemic. The findings from this research indicate that the results presented in this paper are not due to either measurement error or due to unique features of a specific EPU index. Instead, they indicate that there are stable patterns of government expenditure in relation to economic changes.

Using the robustness of this research, and regardless of the substitution of dependent variable for the independent variable, our findings regarding the time-varying characteristics of the shocks caused by EPU on the health and medical industry in China has not changed. The structural findings of the shift from pro-cyclical contraction to counter-cyclical expansion during financial crises and public health crises have similarly remained consistent across all of the robustness tests. Through all of the numerous layers of verification, we establish that the benchmark model results have a high degree of reliability, validity, and internal consistency.

### 3.4 Heterogeneity analysis

This study will focus on understanding how EPU will affect medical spending within regions of China. To do this we will look at different regions of China that have different geographic and economic characteristics and how those differences impact local spending on health care. We have developed our method of classification based on previous research in which scholars use different methods to describe how fiscally decentralized regions are (21) and how hierarchical differences exist among cities in China (22). Therefore, our key hypothesis is that fiscal autonomy and economic strength provide regions with either more or less sensitivity to EPU shocks.

In selecting provinces for analysis, we established two groups based on fiscal capacity and economic development. One group includes provinces with very strong fiscal resources and higher levels of economic development, specifically Beijing, Guangdong, Jiangsu, and Shanghai. All four provinces are located in the southeastern region of China, which is known as the economic powerhouse of the entire country due to their large amounts of revenue generated from taxes and their high level of independence in managing their budgets. The other group contains provinces with relatively weak fiscal capabilities such as Fujian, Hebei, Tianjin, and Yunnan. Through examining the impulse response profiles of both provinces, we have identified distinct regulatory mechanisms within medical and health budget expenditures under EPU shocks, and we determined that there are differences between pro and counter-cyclical responses.

This analysis of the heterogeneity of how different provinces responded to COVID-19 employed a modified approach to the time series than what was used for the initial analysis presented. Where the initial study to establish baseline information for this report used an integrated national dataset that was comprehensive and quarter-over-quarter for each of the years 2007 through to 2025, when conducting a regional analysis, it became apparent that the integrated nature of the data would have to be truncated in some regions to accommodate for the statistical inconsistencies created by the COVID-19 pandemic. In 2020, the COVID-19 pandemic resulted in disruptions to statistical reporting through some provincial governments’ reporting processes with many provincial governments unable to provide statistical information for many months at a time during this period and, also, some of the information they did report contained a number of inaccuracies stemming from the way that the COVID-19 lockdowns and emergency funding engaged local governments. The pandemic had the effect of producing an asymmetrical exogenous shock that overwhelmed the normal business cycle resulting in a potential masking of the structural differences between the provinces that are intended to be examined in this analysis or any future studies of similar type. Therefore, in order to enable examination of the structural differences between provinces while also being cognizant of limitations in data availability and reliability, the sample time period included for the heterogeneity analysis has been revised to include only the pre-pandemic time period (2009 to 2019). Through this modification of the sample period the results of the heterogeneity analysis will more accurately depict the typical structural fiscal behavior that would be expected from provinces under average fluctuations in uncertainty, and would eliminate outlier extremes that were produced as a consequence of the COVID-19 public health emergency in 2020.

To validate the classification of these regions and demonstrate the disparity in fiscal resources, we analyzed the General Public Budget Expenditure data. For financially robust regions such as Beijing, Guangdong, Jiangsu, and Shanghai, their general budget expenditures consistently ranked within the top third nationally from 2009 to 2019. Specifically, based on 2017 provincial general public budget expenditure data (in billions of yuan), the performance of the eight provinces examined in this study was as follows: Beijing (682.453 billion yuan), Guangdong (1503.748 billion yuan), Jiangsu (1062.103 billion yuan), and Shanghai (754.762 billion yuan) all significantly outperformed most other provinces during the same period. These figures are significantly higher than the vast majority of provinces in China during the same period, indicating a massive reservoir of fiscal resources. In sharp contrast, the comparative group demonstrated markedly lower expenditure scales. For the same year, Fujian (468.415 billion yuan), Hebei (663.918 billion yuan), Tianjin (328.254 billion yuan), and Yunnan (571.297 billion yuan), indicating a notably lower overall expenditure scale. The discrepancy is particularly evident when comparing economic hubs like Shanghai to counterparts like Tianjin or Fujian. This substantial gap in fiscal scale confirms that the grouping method is statistically sound and accurately reflects the dichotomy between resource-rich and resource-constrained regions.

From an institutional perspective, the heterogeneous responses across regions can be attributed to China’s fiscal decentralization system. Under the principle of “hierarchical management and hierarchical burden-sharing”, local governments bear the primary responsibility for healthcare financing within their jurisdictions. Regions with strong fiscal capacity, such as have established comprehensive compensation mechanisms for public hospitals and robust budget management systems, enabling them to maintain counter-cyclical healthcare spending during EPU shocks.

China’s fiscal decentralization system is the basis for understanding the varied responses by all regions to China’s economic challenges from an Institutional Perspective. The first component of the fiscal system is that local governments are the principal providers for healthcare financing in their region under “named responsibilities for each level of government or vertical management system and the distribution of burden-sharing”. For instance, the regions that have the greatest fiscal capacity, such as Beijing,^1^ Guangdong,^2^ Jiangsu,^3^ and Shanghai,^4^ have established a complete compensation program for the municipality or region’s hospitals and a complete accounting system for the budget to support counter-cyclical healthcare spending on public health due to health system misfortunes in their regions during EPU.

**Figure 8:**
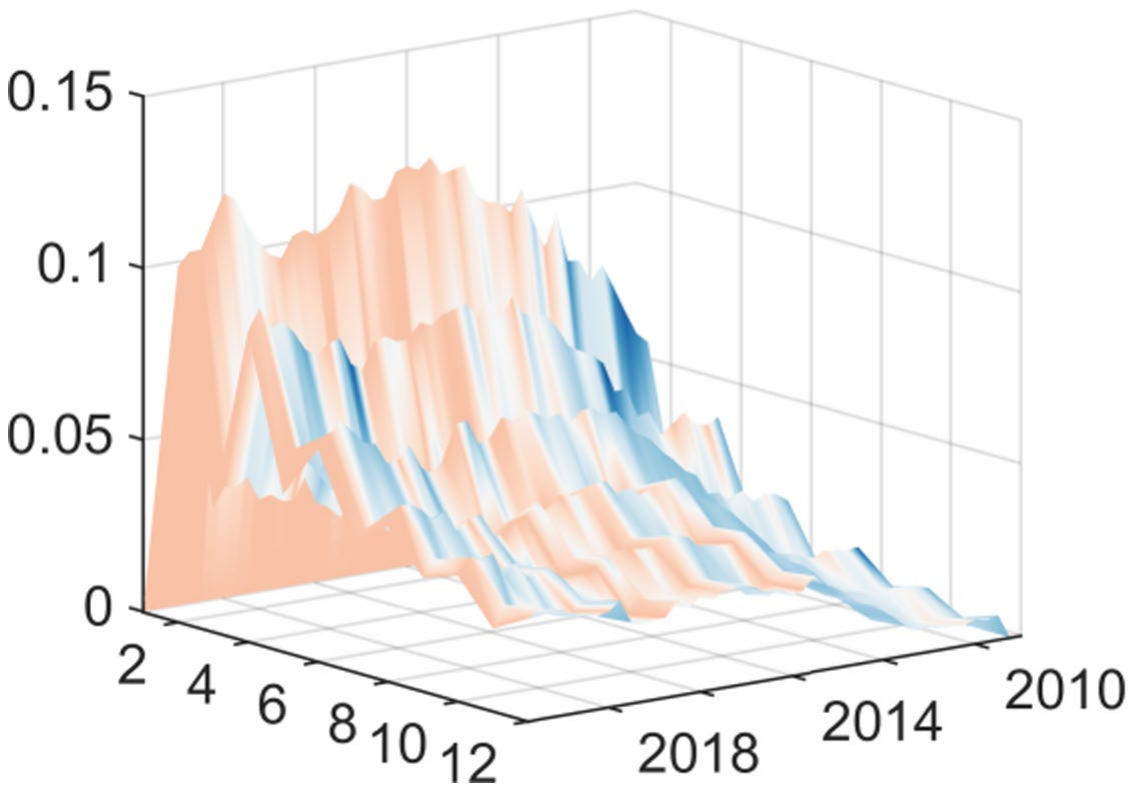
3D time series surface plot of Beijing.

**Figure 9:**
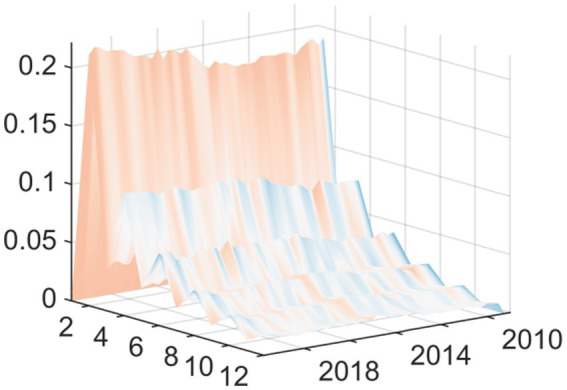
3D time series surface plot of Guangdong.

**Figure 10:**
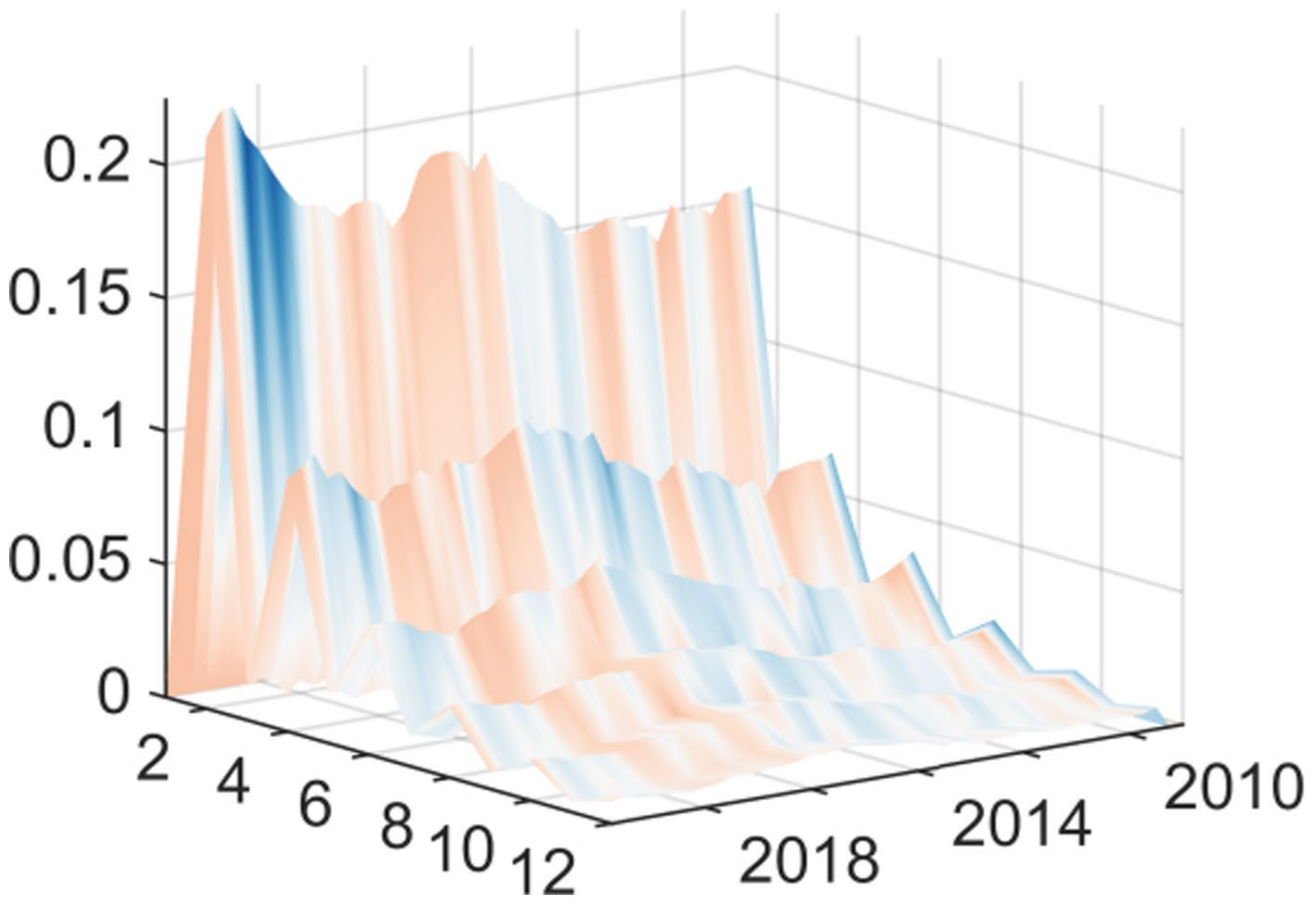
3D time series surface plot of Jiangsu.

**Figure 11:**
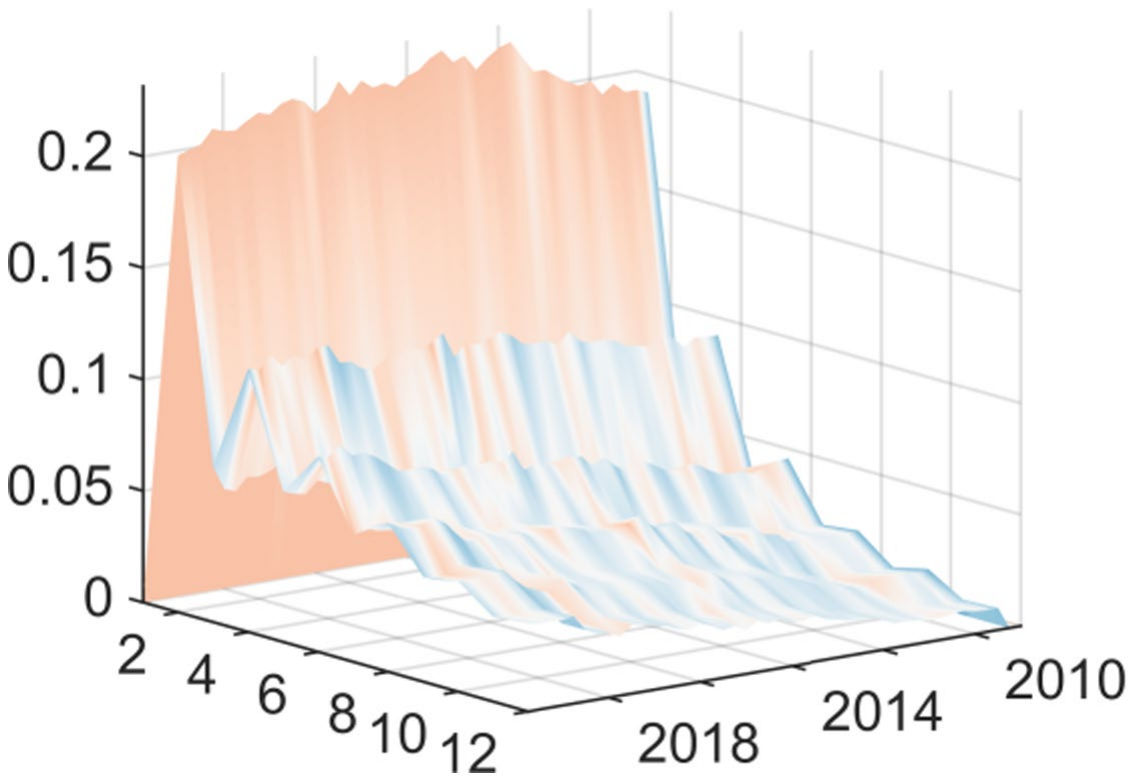
3D time series surface plot of Shanghai.

The results of the pulse response function strongly prove how financial capacity determines the policy response. For areas with strong financial strength such as Beijing, Guangdong, Jiangsu and Shanghai, the response of medical and health expenditure to the impact of economic policy uncertainty shows a clear pattern. Specifically, in the face of the impact of positive economic policy uncertainty, especially in the early stage, these areas in good financial condition show a significant positive response, or, in the worst case, only a negligible negative response (Figures 8–11). This is in stark contrast to the common cyclical contraction in resource-constrained environments. These economically developed provinces have sufficient financial reserves and a wider tax base. Therefore, when economic policy uncertainty rises, these local governments do not need to take austerity measures. On the contrary, they have both the ability and the political will to implement countercyclical fiscal policies. In the face of external shocks, these governments can use sufficient financial space to maintain or even significantly increase spending on key public welfare areas such as health care. This kind of expenditure has a dual role: first, it ensures the continuity of public services; second, it acts as a signal to stabilize social expectations. By protecting the medical and health sectors from fluctuations in economic uncertainty, these regions show significant countercyclical adjustment characteristics.

Conversely, regions with low fiscal capacity will encounter structural hindrances, such as Fujian,^5^ Hebei,^6^ Tianjin,^7^ and Yunnan,^8^ meaning that they will have relatively slow growth in fiscal revenue, have a narrow tax base and, with significant dependency of the revenues on transfer payments from upper levels of government, there are limited fiscal buffers to absorb external shocks.

**Figure 12:**
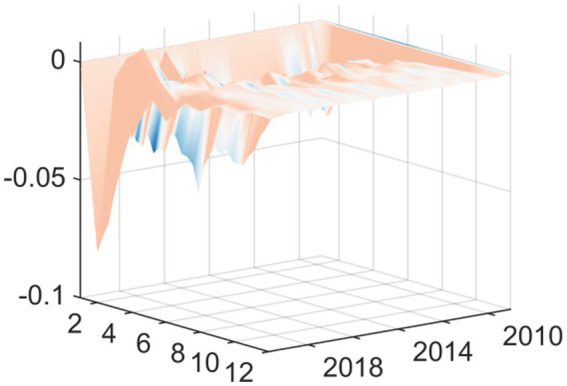
3D time series surface plot of Fujian.

**Figure 13:**
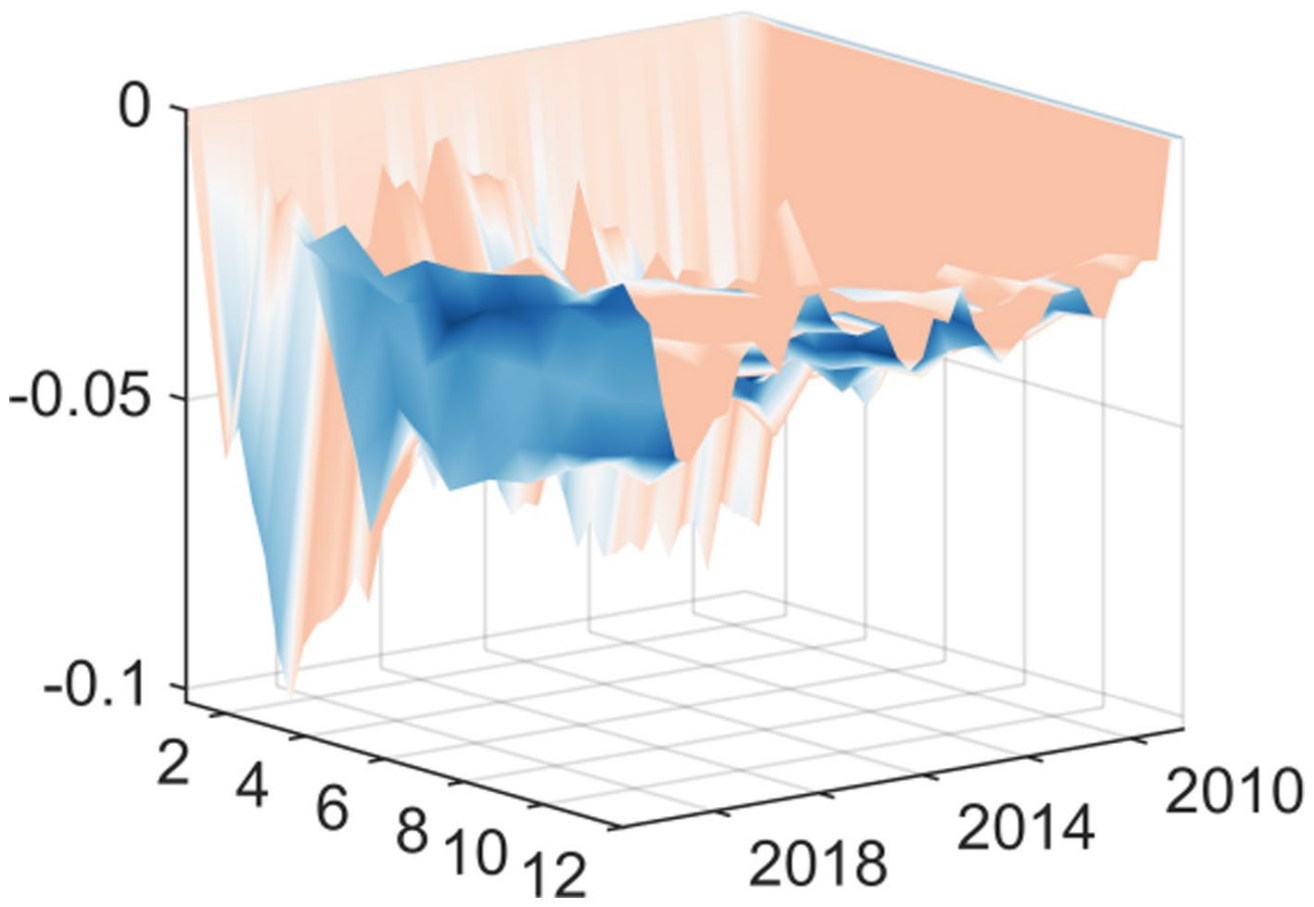
3D time series surface plot Hebei.

**Figure 14:**
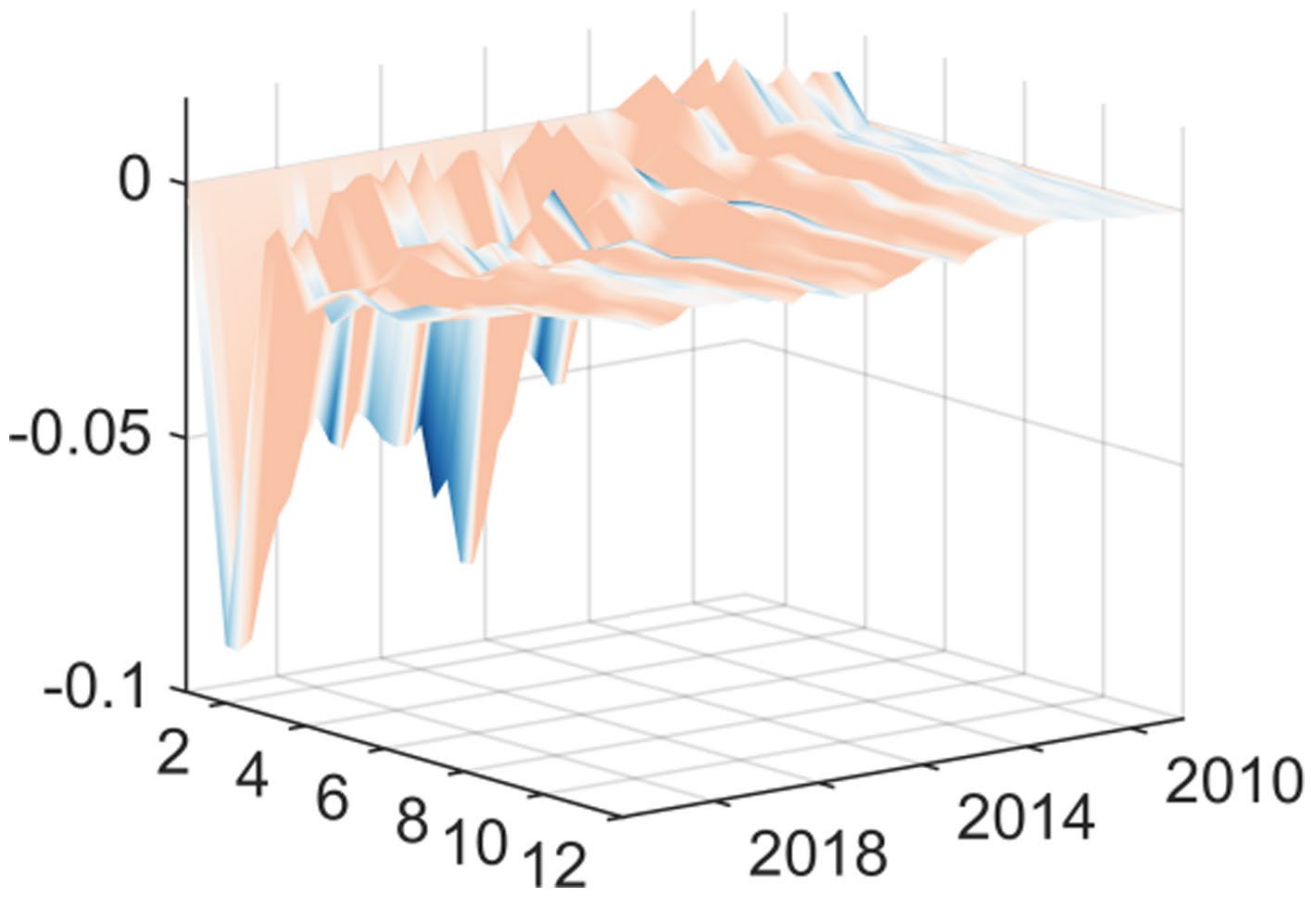
3D Time Series Surface Plot of Tianjin.

**Figure 15:**
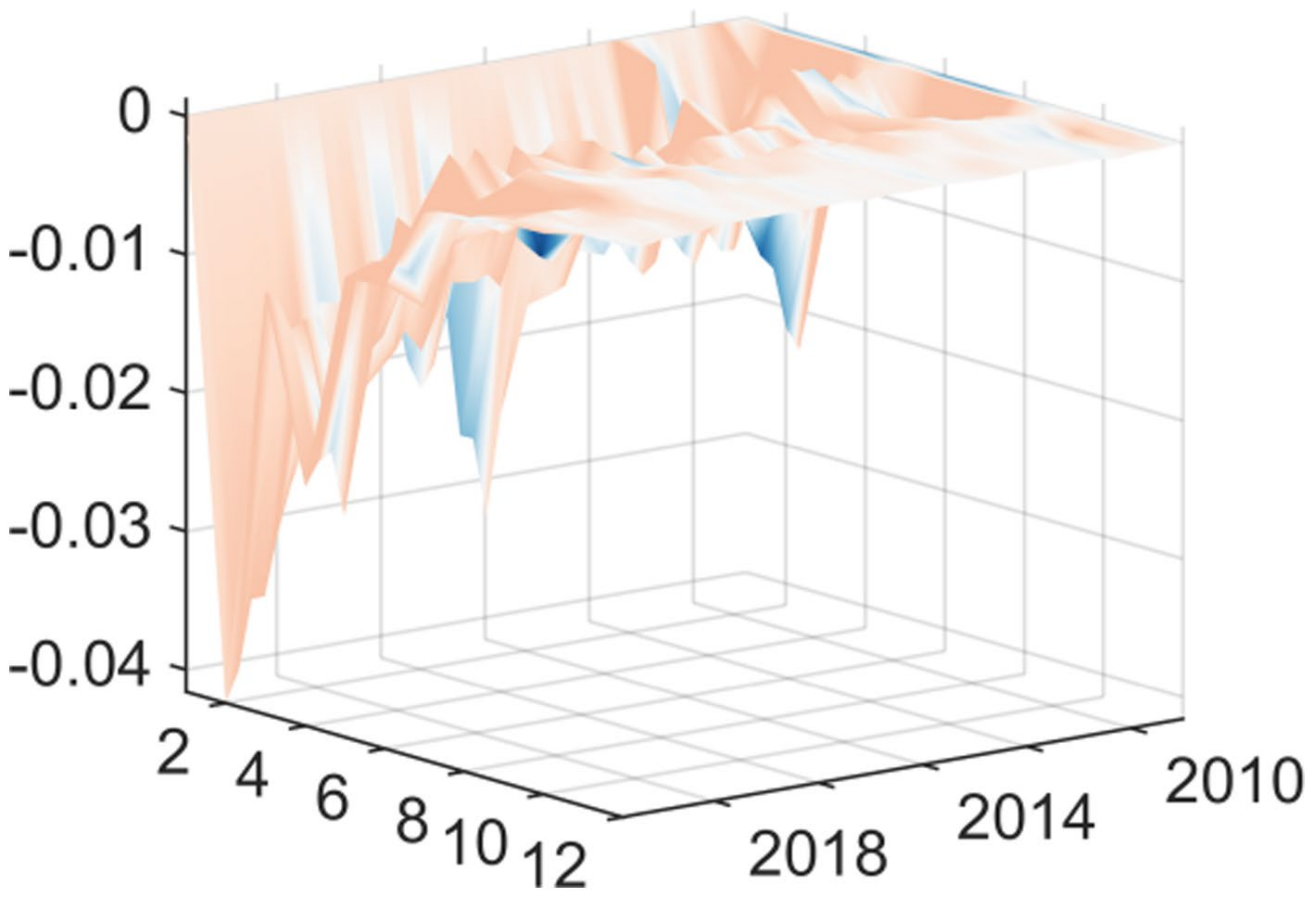
3D time series surface plot of Yunnan.

From the perspective of regional heterogeneity, Fujian, Hebei, Tianjin, and Yunnan exhibit more pronounced and persistent negative responses in healthcare fiscal expenditures when facing economic policy uncertainty shocks. Particularly in the short term following such shocks, their expenditure behavior displays typical procyclical characteristics (Figures 12–15). These regions commonly face structural challenges including relatively sluggish fiscal revenue growth, a narrow tax base, and high dependence on transfer payments, resulting in weaker fiscal buffers when responding to external shocks. According to Guan and Fu (23), there is an increased likelihood that local governments will discontinue public service expenditures when there is greater fiscal pressure on those governments. When local governments experience fiscal vertical imbalances (local fiscal capacity not matching expenditure responsibility), they will have less regard for public services. This is particularly true in financially weaker regions. When the Economic Policy Uncertainty (EPU) increases causing macroeconomic tightening (tightening of the economy) and fiscal revenue pressures, local governments will be under several imperatives to protect wages, operational functions, and basic public welfare and thus must prioritize expenditures. During this prioritization of expenditures, spending for health care is often cut first because health care expenditures are relatively less rigidly budgeted, there are lower political costs associated with cutting hard expenditures, and revenues are uncertain. In summary, rising EPU makes health care expenditures in these regions more susceptible to cuts. This aligns with Zhang et al. (24) conclusion, which indicates that local governments prioritize their mandatory expenditures at the time of fiscal pressure while also cutting their optional (soft) expenditures like public service expenditures. Therefore, a local government’s behavior will reflect a behavior of reducing soft expenditures before hard expenditures. Fiscal pressures affect many local governments by creating clear attempts to reduce the amount of revenue they collect. Because of limited revenue growth, local governments are resorting to implicit reductions in health services through denial of reimbursement requests, deferring reimbursements, reducing health budgets, and changing the way they account for the expenses of health services to provide funding to solve their immediate fiscal problems. This covert reduction in funding for health services creates a situation where the long-term sustainability of these services is compromised, thereby worsening the disparity between different regions with respect to the delivery of public services. Weak fiscal capacity creates both limits on the ability of local governments to spend and limits on the ability of local governments to use regulation to provide counter-cyclical regulation. The fiscal decentralization system allows local governments to have a fair amount of autonomy over how they spend their funds. However, this autonomy becomes a structural constraint when the local government experiences tight fiscal conditions. Without adequate resources to smooth the effects of economic cycles, local governments will often attempt to reduce non-core spending as a way to solve their fiscal problems created by uncertainty. This will create a vicious cycle for the local governments.

The regional heterogeneity aspect of the impulse response analysis illustrates a distinct difference between how provinces respond to EPU shocks according to their fiscal capacities. Specifically, provinces with low fiscal capacity, i.e., Fujian, Hebei, Tianjin, and Yunnan, behave differently when compared to high fiscal capacity provinces, when faced with EPU shocks. The medical and health fiscal expenditures of provinces with lower fiscal capacity respond negatively to EPU shocks over a long-term basis (Figures 12–15); this is particularly apparent immediately after an EPU shock, and the expenditure behavior is typically pro-cyclical. Therefore, as the macroeconomic environment deteriorates and uncertainty increases, lower fiscal capacity governments will decrease expenditures, which has the effect of exacerbating rather than mitigating the economic cycle.

This pro-cyclical downturn is the result of the built-in weaknesses within the public finance systems of the respective regions mentioned. These provinces’ economies are not as diverse or strong as those in the other provinces, and therefore they generally have deeply rooted structural problems. For example, there is relatively slow growth of fiscal revenue, a limited number of tax sources, and a substantial dependence upon transfer payments from the central government. These characteristics in aggregate do not provide a sufficient “fiscal buffer” to withstand the effects of a given external shock; that is, when the given external shocks occur, there exists no accumulated savings from which these regions can draw on to absorb the effects of the external shocks. As a consequence, macroeconomic fluctuations have a significant impact on these regions’ fiscal sustainability; as uncertainty grows, taxes often decline or level off (therefore producing immediate liquidity concerns), forcing the local government to make difficult resource allocation choices.

Taken together, the variation in the responses of regions to fiscal uncertainty is primarily attributable to the differences in the fiscal capacities across regions, which is an issue caused by the institutional arrangements of the fiscal decentralisation framework in China, which creates a competition among local governments that leads to the prioritization of social welfare expenditures. In addition, the fiscal pressures faced by fiscally weak regions create the motivation to decrease public service expenditure. This provides an important institutional framework for understanding why there is so much variability within regions. Regions with strong fiscal capacities will mitigate the effects of the uncertainty shocks by maintaining or increasing healthcare expenditures, as they are characterized by strong countercyclical tendencies. Evidence from prior research indicates that increased fiscal autonomy provides greater resilience for local governments to expend on social welfare during times of economic disruption and maintain the provision of essential public services to their residents (25). Conversely, fiscally weak regions are subject to budgetary constraints that create the greatest levels of vulnerability to a negative effect of the uncertainty capital shocks upon healthcare spending. Empirical studies have shown that local governments tend to expend their budgeted expenditures to promote economic growth while they are subject to fiscal pressures, and tend to prioritize expenditures related to social welfare, thus producing behaviors that exhibit characteristically pro-cyclical patterns (13). Therefore, the characteristics of regional fiscal strength play an important moderating role in the regional responses to economic policy uncertainty. Recent studies further confirm that increasing general transfer payments can effectively relieve local financial pressure and significantly enhance the stability of basic public service expenditures such as health care (26). This provides an important policy inspiration for improving the financial transfer payment system and enhancing the financial resilience of underdeveloped areas. Therefore, the strength of financial capacity directly determines whether local governments can effectively cushion external shocks through financial expenditure measures.

## 4 Research findings and implications

### 4.1 Research findings

Using quarterly Chinese data (2007–2025) and the SV-TVP-FAVAR model, this study systematically examines the dynamic impact of economic policy uncertainty on China’s healthcare fiscal expenditures. It also investigates, for the first time at the provincial level, how local fiscal heterogeneity influences this transmission mechanism. Key findings include: First, EPU’s effect on healthcare fiscal expenditures exhibits significant time-varying characteristics and scenario dependence. In contrast, during the early stages of the 2020 COVID-19 pandemic, governments adopted expansionary fiscal policies to improve emergency response capabilities in the face of a sudden public health emergency. The counter-cyclical nature of EPU and healthcare expenditure movement illustrated how EPU impacts depend on crisis nature and policies being applied to address it as the two phenomena moved together.

The findings reveal how regional fiscal capacity moderates EPU shocks. Regions exhibiting strong fiscal resilience showed the greatest capacity to absorb the EPU shock using counter-cyclical spending patterns whereas regions exhibiting weaker fiscal capacity faced many budgetary constraints; therefore, their healthcare expenditure was significantly more susceptible to crowd-out effects as EPU increased while having pronounced pro-cyclical patterns of spending. In addition, results of the three-dimensional impulse response analysis indicate how EPU’s effects on an economy change both in strength and duration over time. For example, during normal economic conditions, there is moderate impact; however, during extreme economic disruptions, impacts become much greater and last longer than they would during normal times, suggesting an increased level of economic vulnerability to extreme EPU shocks.

This study’s results have significant ramifications for the Chinese healthcare system. Finding “pro-cyclical vulnerability,” which has been identified within those regions of China with weak local fiscal capacity, shows how EPU shocks can negatively impact the continuity of access to important medical services while worsening regional health disparities during periods of decreased economic activity. Further, it demonstrates how over-reliance on local fiscal health undermines the ability of China’s current healthcare system to provide a base level of public health on a universal basis.

### 4.2 Policy implications

The world has entered a new era of turbulence and transformation. Factors such as geopolitical conflicts, the resurgence of trade protectionism, and divergent global economic recoveries have led to macroeconomic policy uncertainties becoming prolonged, complex, and normalized. Domestically, amid economic restructuring and sustained pressure on land-related fiscal revenues, local governments face a structural tension between slowing revenue growth and rigid social welfare expenditures. Particularly in public health, the post-pandemic era’s demand for seamless transition between routine and emergency care underscores the strategic and foundational importance of healthcare financing. Given that China’s healthcare expenditures have demonstrated strong countercyclical stabilizing effects during public health crises, while regional disparities in fiscal resilience significantly impact policy transmission efficiency, the government can implement institutional adjustments to further strengthen the strategic positioning of healthcare fiscal investments. This involves establishing a long-term healthcare investment mechanism aligned with fiscal sustainability. The focus of these efforts should be on creating a systematic approach to optimizing specific fiscal policy instruments that are utilized in the healthcare system. The main components of this fiscal policy should be: (1) the central government must increase its structural share of funding for core public health services, thereby alleviating the heavy financial burden placed on fiscally vulnerable local governments and preventing them from having to impose pro-cyclical cuts to their healthcare budgets during an economic downturn; and (2) the system of transfer payments must be improved by implementing purpose-specific transfers. General-purpose transfers should be significantly increased in order to create a permanent “fiscal buffer” for economically disadvantaged parts of the country to ensure the continuity of routine medical service regardless of the occurrence of an EPU shock, while at the same time the Special transfer payments and Direct Benefit funding mechanisms should be established for use in emergency situations. This will assure that if a sudden public health emergency occurs, central emergency funds can be quickly and directly allocated to the health setup of those facilities located at the community level, thus ensuring that there will be an immediate counter-cyclical expansion in resources. The empirical research in this paper enables us to confirm the operational and effective nature of these predetermined programs. First, we can verify the effectiveness of the expansion of general transfer payments, through the ‘counter-cycle’ evidenced by the heterogeneity analysis. Just as fiscally resilient areas like Beijing and Guangdong use their large reserves to manage health care expenditure during EPU shocks, providing permanent fiscal buffers to those vulnerable areas will neutralize their structural ‘pro-cyclical vulnerability’—as demonstrated in an analysis by Yu et al. (26). Secondly, from our three-dimensional impulse response research, we find that rapid, unhindered fiscal injections produce a high level of counter-cyclical expansion. By institutionalizing these direct channels, we ensure the successful 2020 expansions will be reproduced systematically in future crises and provide means to bypass local funding bottlenecks. Finally, by moving the responsibility for structural expenditure upwards, we will eliminate the root cause of the ‘crowding-out effect’ that we saw during the 2008 financial crisis while continuing to maintain the protection of basic healthcare welfare from being sacrificed in favor of local economic stimulus.

Simultaneously, policy efforts should prioritize boosting domestic demand and invigorating market entities. Creating a more open international commerce will lead to the location and creation of different international marketplaces that will provide opportunities for the enactment of domestic fiscal policy actions. In addition, with markets being more accessible, countries will have a better opportunity to withstand external shocks, which will facilitate a more equitable and quality level of development for social programs including healthcare. Countries also need to improve their cross-boundary co-ordination and communication through the development of better processes for transmitting fiscal policy actions and responding to emergency situations to improve their transmission and response capacities. These improvements to systems and capacities will contribute positively to the overall stability and resiliency of the Chinese healthcare system to effectively deal with and respond to major public health challenges and will be instrumental in ensuring the overall long-term health of the Chinese economy and society.

## 5 Discussion

The empirical findings of this research study suggest that EPU in China is not an absolute and constant force, but displays considerable heterogeneity in time and situation. The direction and magnitude of the effect of EPU on China’s fiscal expenditure for healthcare is influenced primarily by the type of crisis occurring in China. During the Global Financial Crisis of 2008, EPU created a procyclical relationship for healthcare expenditures while during the COVID-19 pandemic in 2020, the association between EPU and healthcare spending was countercyclical. Therefore, this is an important finding as it notes the existing gap in previous research regarding the need for dynamic and specific context analyses to determine the magnitude and direction of EPU’s effects, rather than relying upon static or average estimates.

The findings from the study develop or build upon prior studies on EPU and its influence on fiscal behavior but take the knowledge gained about these two concepts further by considering their significant economic effects on businesses and people, beyond just confirming that EPU has been identified as having an impact and is correlated with fiscal behavior. The economic impact of EPU’s influence differs from public to private sector businesses. In private sector businesses EPU creates uncertainty, which directs businesses to utilize their resources more cautiously and slow down investment (2, 4). Conversely, in the public finance domain, the economic impacts of EPU depend on the utility function of the government and the crisis typology. The pro-cyclical tightening of local government budgets in 2008 illustrates this dynamic, as evidenced by the ‘crowding-out’ of public health services, since local governments facing traditional macroeconomic liquidity constraints have to view public health as a ‘soft’ welfare expenditure that can be treated as compressible.

The majority of these structural reform responses to the financial crisis were pro-cyclical, but in 2020, counter-cyclical expansionary measures were enacted, so pro-cyclical fiscal behavior has been overridden by counter-cyclical fiscal behavior. The failure of healthcare to operate as an elastic resource because of systemic threat to society means it now functions as a ‘fixed security obligation’ which, in turn, creates a non-negotiable obligation for emergency resource mobilization. This suggests that there is a hierarchical structure in the distribution of fiscal resources based on the nature of the impact of the crisis on the overall economic environment. Furthermore, the finding that the strong fiscal regions of the country are exhibiting counter-cyclical resilience while the weak fiscal regions of the country are exhibiting pro-cyclical vulnerability adds to and further delineates the findings within the studies of Bai et al. (3) concerning spatial spillover effects. By extension, this suggests that EPU is not simply a quantitative shock to the economy, but rather it is a structural reallocation mechanism or process whose dynamics are impacted significantly by the relative local capacity of the local area to absorb the various kinds of shocks.

Even though this study reveals many insights, several limitations of this study should be acknowledged. The first limitation is that even though the SV-TVP-FAVAR model is able to recreate dynamic interactions very well, the extracted common factors that are reflective of major economic events will not have any immediate economic interpretation associated with them. Future studies may be able to integrate these types of structural shocks into their frameworks. Secondly, the provincial heterogeneity analysis was only performed for the 2009–2019 time frame due to a lack of available data; therefore, it is not possible to directly compare regional reactions to the unique 2020 pandemic shock under the same framework. It is therefore impossible to determine whether or not regional disparities have grown during the last major economic crisis. Lastly, the study looks primarily at the total healthcare fiscal expenditure response; the details of how healthcare dollars were allocated at the micro level and their ultimate effects on healthcare quality and access remain issues that warrant further investigation using more detailed data.

Going forward, future research can be significantly expanded in the following three dimensions to build upon the findings of this study. First, looking at the spatial and granular levels, future research should focus on developing localized EPU indices at the city or county level. The integration of these highly granular indices with spatial econometric models will enable researchers to delve deeper into the dynamics of intra-regional disparities and the spatial spillover patterns of local fiscal responses to uncertainty. Second, future research should focus on the micro and outcome aspects of the study and use micro-data to analyze the different categories of spending within health care. Linking specific budget items to certain public health outcomes like mortality or disease allows researchers to assess the efficiency and equity of governmental response to crises. Third, concerning the typology of crises, future research should aim to broaden the scope of this analytical framework to different types of uncertainty shocks. The comparative study of the fiscal consequences of endogenous economic adjustments and exogenous structural shocks will help formulate a comprehensive theory on government fiscal choices.

In order to build on the results of this research, future studies could greatly enhance the understanding of the area through further research in three areas. The first is regarding To develop EPU indexes at the local level. Once the very granular index is built, combining it with spatial econometrics will help researchers understand the dynamics behind intra-regional differences and to capture spatial spillover effects due to the uncertainty surrounding a local entity’s fiscal response. Second, in terms of micro-analysis, researchers may use micro-data regarding public health to evaluate the efficiency and effectiveness of government fiscal responses to uncertainty. For example, determining how much preventive public health expenditures versus direct public hospital subsidies contribute to public health outcomes. Finally, regarding the types of crises that affect decision making, future studies could use a larger range of uncertainty shocks to enhance the analytical framework described in this paper. Researchers may, for example, systematically compare the effects of endogenous economic shocks to exogenous and systemic shocks on the fiscal impacts of government decision making.

## Data Availability

Publicly available datasets were analyzed in this study. This data can be found at: https://www.wind.com.cn/portal/zh/EDB/index.html.

## Footnotes

1
https://china.huanqiu.com/article/9CaKrnJWjR0

2
https://news.hexun.com/2019-11-19/199318845.html

3
https://www.qunzh.com/qzxlk/jczx/2017/201716/202011/t20201104_82006.html

4
https://www.shanghai.gov.cn/nw30612/20200820/0001-30612_31927.html

5
https://czt.fujian.gov.cn/zwgk/zfxxgk/fdzdgknr/czzjgl/zjglbf/202301/t20230131_6100714.htm

6
https://m.mof.gov.cn/czxw/201810/t20181015_3045666.htm

7
https://www.tj.gov.cn/zwgk/zcjd/202005/t20200519_2376798.html

8
http://union.china.com.cn/txt/2019-10/15/content_40921635.html

## Appendix Table 1 Descriptive statistical analysis

**Table 1:**

Variable	Number of observations	Mean	Standard Err.	Kurt.	Skew.	Min.	Max.
CEPU	74	123.47	2.39	0.19	0.28	38.20	281.95
HCE	74	36551.99	2270.90	−1.04	0.37	15234.90	65841.39
HCE_Beijing_	44	1529.39	111.90	−0.56	−0.06	5.20	2964.81
HCE_Guangdong_	44	2987.01	239.74	−0.71	0.18	6.01	6126.41
HCE_Jiangsu_	44	2460.87	181.58	−0.80	−0.19	5.74	4459.17
HCE_Shanghai_	44	1438.98	104.57	−0.35	−0.28	5.06	2532.68
HCE_Fujian_	44	930.87	69.10	−0.81	−0.12	4.46	1699.13
HCE_Hebei_	44	1583.11	112.76	−0.40	−0.40	5.17	2939.94
HCE_Tianjin_	44	600.56	38.89	−0.46	−0.50	4.21	973.51
HCE_Yunnan_	44	973.07	72.49	−0.70	−0.01	4.55	1801.89
